# Association between Circulating Fibroblast Growth Factor 23, α-Klotho, and the Left Ventricular Ejection Fraction and Left Ventricular Mass in Cardiology Inpatients

**DOI:** 10.1371/journal.pone.0073184

**Published:** 2013-09-09

**Authors:** Kensaku Shibata, Shu-ichi Fujita, Hideaki Morita, Yusuke Okamoto, Koichi Sohmiya, Masaaki Hoshiga, Nobukazu Ishizaka

**Affiliations:** Department of Cardiology, Osaka Medical College, Osaka, Japan; Universidade de São Paulo, Brazil

## Abstract

**Background:**

Fibroblast growth factor 23 (FGF23), with its co-receptor Klotho, plays a crucial role in phosphate metabolism. Several recent studies suggested that circulating FGF23 and α-Klotho concentrations might be related to cardiovascular abnormalities in patients with advanced renal failure.

**Purpose:**

Using data from 100 cardiology inpatients who were not undergoing chronic hemodialysis, the association of circulating levels of FGF23, α-Klotho, and other calcium-phosphate metabolism-related parameters with the left ventricular ejection fraction (LVEF) and left ventricular mass (LVM) was analyzed.

**Methods and Results:**

LVEF was measured using the modified Simpson method for apical 4-chamber LV images and the LVM index (LVMI) was calculated by dividing the LVM by body surface area. Univariate analysis showed that log transformed FGF23, but not that of α-Klotho, was significantly associated with LVEF and LVMI with a standardized beta of −0.35 (P<0.001) and 0.26 (P<0.05), respectively. After adjusting for age, sex, estimated glomerular filtration rate, and serum concentrations of intact parathyroid hormone, and 25-hydroxyvitamin D as covariates into the statistical model, log-transformed FGF23 was found to be a statistically positive predictor for decreased left ventricular function and left ventricular hypertrophy.

**Conclusions:**

In cardiology department inpatients, circulating FGF23 concentrations were found to be associated with the left ventricular mass and LVEF independent of renal function and other calcium-phosphate metabolism-related parameters. Whether modulation of circulating FGF23 levels would improve cardiac outcome in such a high risk population awaits further investigation.

## Introduction

Fibroblast growth factor 23 (FGF23), the most recently identified molecule in the FGF family [1], plays a crucial role in phosphate metabolism, in association with parathyroid hormone (PTH) and vitamin D, by mobilizing sodium-phosphate co-transporters in coordination with Klotho [2], [3], a transmembrane protein that has anti-aging properties [4]. Recent studies have shown an association between circulating FGF23 levels and pathologic cardiovascular conditions, including left ventricular hypertrophy [5], [6], and vascular endothelial dysfunction [7]. Such associations were investigated mainly in patients with chronic kidney disease [8], [9], and in community-dwelling people, albeit in fewer studies [10]. Circulating FGF23 levels are known to be elevated in subjects with severe renal failure, presumably acting against phosphate retention on such occasions [11]. Considering that cardiovascular events are increased in patients with a low estimated glomerular filtration rate (eGFR) [12], the possibility exists that increased FGF23 levels mediate an adverse cardiovascular outcome among patients with end-stage renal disease. Increasingly, clinical and experimental studies have tried to verify this hypothesis. It has been elucidated that Klotho, which is expressed in renal tubular epithelial cells, acts as an obligate co-receptor for FGF23 when FGF23 inhibits proximal tubular phosphate reabsorption leading to a reduction in the serum phosphate level [13]. Although a sensitive and specific assay for circulating Klotho (α-Klotho) has become available recently [14], the relationship between Klotho and the cardiovascular system appears to have been less extensively studied; however, the relationship of *klotho* gene polymorphisms and the serum soluble Klotho (α-Klotho) concentration with arteriosclerosis has been suggested in some studies [9], [15].

Epidemiological and clinical studies showed that there may be associations between calcium-phosphorus metabolism-related factors other than FGF23/α-Klotho, such as calcium (Ca), inorganic phosphate (IP), PTH, 25-hydroxyvitamin D [25(OH)D], and cardiovascular risk [16], [17], [18], [19]. To this end, we have investigated whether circulating levels of FGF23 and α-Klotho were associated with the left ventricular ejection fraction (LVEF) and left ventricular hypertrophy independent of these other calcium-phosphorus metabolism-related factors in cardiovascular inpatients, a population at high risk for cardiac abnormalities.

## Methods

### Ethics Statement

Written informed consent was obtained from all patients or their guardians. The study was approved by Local Ethics Committee at the Osaka Medical College and conducted in accordance to the Declaration of Helsinki.

### Study Population

The current retrospective study has been approved by the Ethics Committee of Osaka Medical College. Between 2012 January and 2012 December, 102 patients gave informed consent were included in this study in whom sufficient clinical and echocardiographic information, in addition to serum FGF23 and α-Klotho concentrations and other calcium-phosphate metabolism-related parameters were available. Two of these patients were found to be undergoing chronic hemodialysis. Because chronic hemodialysis may substantially affect the serum levels of α-Klotho and FGF23 [20], [21], we excluded these two patients from the study, and thus, total of 100 patients were enrolled in the current study.

### Laboratory Analysis

Blood samples were collected in the morning after an overnight fast. Aliquots of serum and plasma were immediately obtained and stored at -80 degrees until use. Serum level of soluble α-Klotho was measured using a solid-phase sandwich enzyme-linked immunosorbent assay (ELISA) (Immuno-Biological Laboratories, Gunma, Japan) [14]. Serum intact FGF23 was measured using a two-step FGF23 enzyme immunoassay (ELISA) kit (Kainos Laboratories Inc., Tokyo, Japan) according to the manufacturer's instructions.

Ca, IP, C-reactive protein (CRP), and B-type natriuretic peptide (BNP) were measured by routine laboratory methods. When serum albumin was 4 mg/dL or lower, serum Ca levels were corrected using the formula [Ca + (4–“serum albumin”)], and designated as corrected Ca (cCa). Serum levels of intact PTH (iPTH), 25(OH)D, and 1,25-dihydroxyvitamin D [1,25(OH)_2_D] were measured by the immunochemical detection method using electrochemiluminescence, competitive protein binding assay, and radioimmunoassay, respectively (Mitsubishi Medience, Tokyo, Japan). The eGFR was calculated by the following Modification of Diet in Renal Disease equation modified for Japanese subjects: eGFR  = 194× (serum creatinine)^–1.094^× (age)^–0.287^ (×0.739, when female) [22].

### Echocardiography

Echocardiographic examinations were performed with a Vivid 7 Dimension equipped with a multi-frequency transducer (GE Healthcare, Vingmed, Norway). Left ventricular (LV) end-diastolic dimension (LVDd), interventricular septal thickness (IVST) and posterior wall thickness (PWT) were measured at end diastole. LV volumes were calculated using the modified Simpson method using the apical 4-chamber view. Low LVEF was defined to be present when LVEF was <50%. For calculation of the LV mass (LVM), we used the formula proposed by Devereux et al. [23] with modification: 0.8×1.04×[(LVDd + IVST + PWT)^3^ – LVDd^3^] + 0.6 [24]. Body surface area (BSA) was calculated using the following formula: (body weight)^0.425^× (height)^0.725^× 0.007184, and then the LVM index (LVMI) was calculated as the ratio of LVM to the BSA. When the LVMI was greater than 118 (men)/108 (women) g/m^2^, LV hypertrophy was defined to be present [25]. Transmitral inflow was recorded using pulsed wave Doppler recordings at the mitral valve leaflet tips in the apical 4-chamber view. The peak velocities of early filling (E) and atrial filling (A) were measured. The average of the early peak diastolic mitral annulus velocity obtained at the septal and lateral annulus measured using pulsed wave tissue Doppler imaging, which was designated e'. Because non-sinus cardiac rhythm or certain technical reasons, data on the E/A ratio and E/e' ratio could be obtained in 76 and 48 patients, respectively.

### Statistical Analysis

Baseline characteristics were assessed with standard descriptive statistics. Data were expressed either as mean ± standard deviation, or median and interquartile range (IQR). Spearman rank correlation test was used to assess the correlation between two variables. Univariate or stepwise multivariate linear regression analysis was used to examine the association between LVMI or LVEF and demographic clinical, and laboratory variables. The calcium-phosphate metabolism-related parameters, except IP, (FGF23, α-Klotho, cCa, intact PTH, 25(OH)D, 1,25(OH)_2_D) were not found to be normally distributed by the Kolmogorov-Smirnov test; therefore these values, and IP, were log-transformed for multivariate regression analysis although α-Klotho and cCa were not found to be completely normalized by log transformation. Multivariable logistic regression analysis was performed to examine the association between log(FGF23) concentrations and low LVEF or LV hypertrophy after adjusting for possible confounding variables including eGFR. Data analysis was performed by IBM SPSS statistics version 21.0 (SPSS, Chicago, IL).

## Results

### Patient Characteristics

Demographic and laboratory data and echocardiographic results of the study subjects are shown in Table 1. Among the 100 patients, ischemic heart disease was the most common condition (Table 1). Moderate to severe heart failure (New York Heart Association grades 3 or 4) was present in 12% of the study patients. Neither FGF23 or α-Klotho concentrations differed according to the use of angiotensin converting enzyme inhibitor or angiotensin II receptor blocker, beta blocker, calcium channel blocker, or statin (data not shown). Among 100 patients enrolled, 30 patients (30%) had eGFR of ≥60 mL/min/1.73 m^2^ and 70 patients (70%) had eGFR of <60 mL/min/1.73 m^2^. Only 7 patients (7%) had eGFR of less than 30 mL/min/m^2^. LVDd and LVEF in more than one-fourth of the patients were less than 55 mm and greater than 50%, respectively, in the current study population (Table 2). FGF23 and α-Klotho concentrations were not significantly different between subjects with eGFR of ≥60 mL/min/1.73 m^2^ and those with eGFR of <60 mL/min/1.73 m^2^.

**Table 1:** Demographic characteristics of the study patients.

	Total study patients				eGFR <60 mL/min/m^2^				eGFR ≥60 mL/min/m^2^				
Variables	(n = 100)				(n = 70)				(n = 30)				P value
Clinical characteristics													
Age	65.4	±	13.0		68.4	±	10.9		58.3	±	14.9		<0.001
Sex (women/men)	27	/	73		10	/	60		17	/	13		<0.001
Body mass index, kg/m^2^	23.1	±	3.1		23.2	±	3.0		23.0	±	3.2		0.740
Systolic blood pressure, mmHg	126.1	±	19.7		126.5	±	19.6		125.1	±	20.1		0.744
Diastolic blood pressure, mmHg	71.6	±	12.4		70.9	±	12.0		73.3	±	13.2		0.364
Pulse rate, bpm	72.3	±	13.7		72.6	±	14.9		71.4	±	10.5		0.695
Past History													
previous PCI	44	(	44.0	)	33	(	47.1	)	11	(	36.7	)	0.333
previous CABG	7	(	7.0	)	4	(	5.7	)	3	(	10.0	)	0.441
Cardiovascular disease													
Ischemic heart disease, n (%)	59	(	59.0	)	43	(	61.4	)	16	(	53.3	)	0.451
Cardiomyopathy, n (%)	10	(	10.0	)	7	(	10.0	)	3	(	10.0	)	1.000
NYHA class III/IV, n (%)	12	(	12.0	)	5	(	7.1	)	7	(	23.3	)	0.022
Aortic aneurysm, n (%)	6	(	6.0	)	5	(	7.1	)	1	(	3.3	)	0.462
Arrythmia, n (%)	22	(	22.0	)	12	(	17.1	)	10	(	33.3	)	0.073
Peripheral artery disease, n (%)	8	(	8.0	)	7	(	10.0	)	1	(	3.3	)	0.260
Valvular heart disease, n (%)	8	(	8.0	)	7	(	10.0	)	1	(	3.3	)	0.260
Cardiac rhythm													
Sinus rhythm, n (%)	7	(	7.0	)	4	(	5.7	)	3	(	10.0	)	0.687
Atrial fibrillation, n (%)	15	(	15.0	)	10	(	14.3	)	5	(	16.7	)	
Pace maker rhythm, n (%)	78	(	78.0	)	56	(	80.0	)	22	(	73.3	)	
Smoking status													
Never, n (%)	33	(	33.0	)	24	(	34.3	)	9	(	30.0	)	0.658
Former, n (%)	50	(	50.0	)	33	(	47.1	)	17	(	56.7	)	
Current, n (%)	17	(	17.0	)	13	(	18.6	)	4	(	13.3	)	
Medication													
ACE inhibitors/ARB, n (%)	51	(	51.0	)	41	(	58.6	)	10	(	33.3	)	0.021
Beta blockers, n (%)	38	(	38.0	)	32	(	45.7	)	6	(	20.0	)	0.015
Calcium channel blockers, n (%)	44	(	44.0	)	35	(	50.0	)	9	(	30.0	)	0.065
Aldosterone antagonist, n (%)	13	(	13.0	)	11	(	15.7	)	2	(	6.7	)	0.218
Diuretics													
Loop, n (%)	18	(	18.0	)	15	(	21.4	)	3	(	10.0	)	0.173
Thiazide, n (%)	5	(	5.0	)	4	(	5.7	)	1	(	3.3	)	0.617
Antidiabetic drugs													
Sulfonylurea, n (%)	10	(	10.0	)	7	(	10.0	)	3	(	10.0	)	1.000
DPP4 inhibitors, n (%)	9	(	9.0	)	6	(	8.6	)	3	(	10.0	)	0.819
Insulin, n (%)	4	(	4.0	)	2	(	2.9	)	2	(	6.7	)	0.373
Others, n (%)	8	(	8.0	)	4	(	5.7	)	4	(	13.3	)	0.198
Lipid lowering drugs													
Statin, n (%)	52	(	52.0	)	39	(	55.7	)	13	(	43.3	)	0.256
Fibrate, n (%)	1	(	1.0	)	0	(	0.0	)	1	(	3.3	)	0.125
Others, n (%)	7	(	7.0	)	6	(	8.6	)	1	(	3.3	)	0.347

PCI, percutaneous coronary intervention; coronary artery bypass graft; ACE, angiotensin converting enzyme; ARB, angiotensin II receptor blocker; DPP4, di-peptidyl peptidase-4. P values are meant to the comparison between eGFR ≥60 mL/min/m^2^ and <60 mL/min/m^2^ groups.

**Table 2:** Laboratory and echocardiographic data.

	Total study patients						eGFR <60 mL/min/m^2^						eGFR ≥60 mL/min/m^2^						
Variables	(n = 100)						(n = 70)						(n = 30)						P value
Laboratory data																			
White blood cell count, ×10^3^/mL	5.93	(	4.92	-	7.15	)	5.99	(	4.87	-	7.25	)	5.86	(	5.07	-	6.63	)	0.860
Hemoglobin, g/dL	13.3	(	12.4	-	14.6	)	13.3	(	12.3	-	14.5	)	13.7	(	12.4	-	14.7	)	0.391
Platelet count, ×10^4^/mL	21.7	(	18.8	-	26.3	)	24.2	(	20.0	-	28.4	)	24.2	(	20.0	-	28.4	)	0.105
Total protein, g/dL	7.0	(	6.7	-	7.4	)	7.0	(	6.8	-	7.3	)	6.9	(	6.7	-	7.5	)	0.967
Albumin, g/dL	4.0	(	3.7	-	4.2	)	3.9	(	3.7	-	4.2	)	4.0	(	3.8	-	4.2	)	0.448
Total cholesterol, mg/dL	178	(	159	-	196	)	175	(	155	-	196	)	185	(	166	-	208	)	0.078
HDL cholesterol, mg/dL	49	(	42	-	63	)	48	(	42	-	58	)	54	(	43	-	68	)	0.408
LDL cholesterol, mg/dL	97	(	78	-	117	)	95	(	76	-	119	)	103	(	84	-	116	)	0.065
Triglycerides	132	(	86	-	176	)	126	(	87	-	21	)	137	(	82	-	189	)	0.321
Blood urea nitrogen	16.0	(	13.3	-	20.0	)	17.0	(	14.0	-	21.0	)	14.5	(	11.0	-	17.0	)	0.008
Creatinine, mg/dL	0.9	(	0.7	-	1.0	)	0.9	(	0.8	-	1.1	)	0.7	(	0.6	-	0.7	)	<0.001
eGFR, mL/min/1.73m^2^	54	(	42	-	64	)	46	(	38	-	55	)	69	(	65	-	77	)	<0.001
Alanine transaminase, IU/L	19	(	14	-	27	)	20	(	16	-	27	)	20	(	16	-	27	)	0.248
Aspartate transaminase, IU/L	22	(	19	-	27	)	21	(	20	-	27	)	23	(	18	-	27	)	0.851
B-type natriuretic peptide, pg/mL	36.8	(	13.5	-	129.0	)	43.1	(	16.1	-	176.2	)	29.4	(	9.8	-	110.8	)	0.175
C-reactive protein, mg/dL	0.09	(	0.03	-	0.23	)	0.09	(	0.03	-	0.20	)	0.09	(	0.05	-	0.25	)	0.676
Corrected calcium, mg/dL	9.2	(	8.9	-	9.4	)	9.2	(	8.9	-	9.4	)	9.1	(	8.9	-	9.4	)	0.561
Inorganic phosphate, mg/dL	3.4	(	3.0	-	3.8	)	3.3	(	3.0	-	3.6	)	3.6	(	3.2	-	4.0	)	0.019
Intact parathyroid hormone, pg/mL	37.5	(	29.3	-	48.8	)	37.5	(	29.8	-	25.5	)	37.5	(	25.8	-	46.5	)	0.638
25(OH) Vitamin D, pg/mL	19.8	(	16.1	-	25.0	)	20.7	(	17.4	-	25.5	)	16.4	(	13.9	-	21.3	)	0.005
1,25(OH)_2_ Vitamin D, pg/mL	59.0	(	41.0	-	80.8	)	59.0	(	41.8	-	86.3	)	54.5	(	40.0	-	72.5	)	0.359
FGF23, pg/mL	68.6	(	42.4	-	127.0	)	74.5	(	42.5	-	151.7	)	64.3	(	41.4	-	82.3	)	0.256
α-Klotho, pg/mL	443.3	(	273.7	-	595.1	)	443.3	(	268.4	-	613.6	)	443.4	(	333.4	-	568.6	)	0.729
Echocardiographic data																			
LVDd, mm	4.9	(	4.6	-	5.4	)	4.9	(	4.7	-	5.4	)	4.8	(	4.4	-	5.3	)	0.142
LVDs, mm	3.2	(	2.8	-	3.8	)	3.1	(	2.7	-	3.6	)	3.1	(	2.7	-	3.6	)	0.362
IVST, mm	1.0	(	0.9	-	1.1	)	1.0	(	0.9	-	1.1	)	0.9	(	0.8	-	1.0	)	0.052
PWT, mm	1.0	(	0.9	-	1.1	)	1.0	(	0.9	-	1.1	)	0.9	(	0.9	-	1.0	)	0.156
LVEF, %	61	(	53	-	65	)	61	(	56	-	65	)	62	(	52	-	66	)	0.804
LVMI, g/m^2^	106	(	86	-	127	)	108	(	87	-	129	)	96	(	83	-	123	)	0.215
E/A (n = 76)	0.8	(	0.6	-	1.1	)	0.8	(	0.6	-	1.1	)	0.8	(	0.7	-	1.1	)	0.562
E/e' (n = 48)	8.8	(	7.4	-	10.6	)	9.4	(	7.6	-	11.9	)	8.2	(	7.1	-	9.3	)	0.284

LVDd, left ventricular diastolic dimension; LVDs, left ventricular systolic dimension; IVST, interventricular septum thickness; PWT, posterior wall thickness; LVEF, left ventricular ejection fraction; LVMI, left ventricular mass index. P values are meant to the comparison between eGFR ≥60 mL/min/m^2^ and <60 mL/min/m^2^ groups.

### Correlation between α-Klotho, FGF23, and Other Clinical and Laboratory Variables

Correlation between FGF23, α-Klotho, and clinical and laboratory parameters were analyzed (Tables 3, 4). FGF23 was found to be correlated negatively with body mass index and hemoglobin concentrations. α-Klotho was significantly correlated negatively with blood urea nitrogen and positively with aspartate aminotransferase levels. eGFR showed borderline significant correlation with FGF23, but not with α-Klotho. We next investigated the association between log-transformed FGF23 and α-Klotho and other calcium-phosphate metabolism-related parameters (Table 5). It was found that log(FGF23) was correlated negatively with log(α-Klotho) and positively with log(IP), and Log(α-Klotho) was positively correlated with log(25(OH)D) and log(1,25(OD)_2_D).

**Table 3:** Spearman's correlation coefficients between FGF23 and various variables.

	FGF23					
	Total study patients		eGFR <60 mL/min/m^2^		eGFR ≥60 mL/min/m^2^	
	(n = 100)		(n = 70)		(n = 30)	
	r	P value	r	P value	r	P value
Age, year	−0.03	###	0.05	###	−0.44	###
Sex, (male = 1)	0.11	###	−0.01	###	0.20	###
Body mass index	−0.20	###	−0.26	###	−0.13	###
Systolic blood pressure	−0.17	###	−0.14	###	−0.26	###
Diastolic blood pressure	−0.17	###	−0.16	###	−0.14	###
Pulse rate	0.02	###	0.18	###	−0.58	###
White blood cell count	−0.04	###	−0.14	###	0.18	###
Hemoglobin	−0.24	###	−0.41	###	0.18	###
Platelet count	0.00	###	0.04	###	−0.02	###
Total protein	0.13	###	0.17	###	0.04	###
Albumin	−0.06	###	−0.16	###	0.19	###
Total cholesterol	−0.01	###	−0.04	###	0.11	###
LDL cholesterol	−0.02	###	−0.07	###	0.12	###
HDL cholesterol	0.06	###	0.14	###	−0.05	###
Triglycerides	−0.05	###	−0.15	###	0.19	###
Blood urea nitrogen	0.19	###	0.24	###	−0.01	###
Creatinine	0.20	###	0.11	###	0.47	###
eGFR	−0.18	###	−0.12	###	−0.29	###
Alanine transaminase	−0.13	###	−0.25	###	0.31	###
Aspartate transaminase	−0.14	###	−0.23	###	0.16	###
B-type natriuretic peptide	0.20	###	0.16	###	0.23	###
C-reactive protein	0.03	###	0.08	###	−0.09	###
Corrected calcium	−0.02	###	−0.04	###	0.03	###
Inorganic phosphate	0.19	###	0.23	###	0.18	###
Intact parathyroid hormone	0.07	###	0.00	###	0.23	###
25(OH) Vitamin D, pg/mL	−0.03	###	−0.07	###	−0.05	###
1,25(OH)_2_ Vitamin D	0.01	###	0.03	###	−0.06	###

	α-Klotho					
	Total study patients		eGFR <60 mL/min/m^2^		eGFR ≥60 mL/min/m^2^	
	(n = 100)		(n = 70)		(n = 30)	
	r	p value	r	p value	r	p value
Age, year	0.04	###	0.07	###	0.00	###
Sex, (male = 1)	0.14	###	0.27	###	0.00	###
Body mass index	0.08	###	0.12	###	0.03	###
Systolic blood pressure	0.16	###	0.16	###	0.23	###
Diastolic blood pressure	0.18	###	0.17	###	0.27	###
Pulse rate	0.13	###	0.04	###	0.41	###
White blood cell count	0.01	###	0.02	###	−0.09	###
Hemoglobin	0.20	###	0.24	###	0.07	###
Platelet count	−0.13	###	−0.09	###	−0.28	###
Total protein	−0.06	###	−0.11	###	0.06	###
Albumin	0.19	###	0.18	###	0.16	###
Total cholesterol	0.04	###	0.05	###	−0.03	###
LDL cholesterol	0.02	###	0.08	###	−0.18	###
HDL cholesterol	0.10	###	0.05	###	0.19	###
Triglycerides	−0.05	###	0.01	###	−0.16	###
Blood urea nitrogen	−0.25	###	−0.33	###	−0.08	###
Creatinine	−0.15	###	−0.21	###	−0.16	###
eGFR	0.15	###	0.20	###	0.24	###
Alanine transaminase	0.15	###	0.22	###	−0.07	###
Aspartate transaminase	0.23	###	0.29	###	0.13	###
B-type natriuretic peptide	−0.20	###	−0.24	###	−0.07	###
C-reactive protein	−0.16	###	−0.08	###	−0.39	###
Corrected calcium	0.09	###	0.05	###	0.13	###
Inorganic phosphate	−0.17	###	−0.23	###	−0.10	###
Intact parathyroid hormone	−0.08	###	0.03	###	−0.30	###
25(OH) Vitamin D, pg/mL	0.16	###	0.10	###	0.34	###
1,25(OH)_2_ Vitamin D	0.17	###	0.17	###	0.10	###
age_2	−0.03	###	−0.03	###	−0.03	###
Sex	0.11	###	0.11	###	0.11	###
BMI	−0.20	###	−0.20	###	−0.20	###
BPs	−0.17	###	−0.17	###	−0.17	###

**Table 4:** Spearman's correlation coefficients between α-Klotho and various variables.

	FGF23					
	Total study patients		eGFR <60 mL/min/m^2^		eGFR ≥60 mL/min/m^2^	
	(n = 100)		(n = 70)		(n = 30)	
	r	P value	r	P value	r	P value
Age, year	−0.03	###	0.05	###	−0.44	###
Sex, (male = 1)	0.11	###	−0.01	###	0.20	###
Body mass index	−0.20	###	−0.26	###	−0.13	###
Systolic blood pressure	−0.17	###	−0.14	###	−0.26	###
Diastolic blood pressure	−0.17	###	−0.16	###	−0.14	###
Pulse rate	0.02	###	0.18	###	−0.58	###
White blood cell count	−0.04	###	−0.14	###	0.18	###
Hemoglobin	−0.24	###	−0.41	###	0.18	###
Platelet count	0.00	###	0.04	###	−0.02	###
Total protein	0.13	###	0.17	###	0.04	###
Albumin	−0.06	###	−0.16	###	0.19	###
Total cholesterol	−0.01	###	−0.04	###	0.11	###
LDL cholesterol	−0.02	###	−0.07	###	0.12	###
HDL cholesterol	0.06	###	0.14	###	−0.05	###
Triglycerides	−0.05	###	−0.15	###	0.19	###
Blood urea nitrogen	0.19	###	0.24	###	−0.01	###
Creatinine	0.20	###	0.11	###	0.47	###
eGFR	−0.18	###	−0.12	###	−0.29	###
Alanine transaminase	−0.13	###	−0.25	###	0.31	###
Aspartate transaminase	−0.14	###	−0.23	###	0.16	###
B-type natriuretic peptide	0.20	###	0.16	###	0.23	###
C-reactive protein	0.03	###	0.08	###	−0.09	###
Corrected calcium	−0.02	###	−0.04	###	0.03	###
Inorganic phosphate	0.19	###	0.23	###	0.18	###
Intact parathyroid hormone	0.07	###	0.00	###	0.23	###
25(OH) Vitamin D, pg/mL	−0.03	###	−0.07	###	−0.05	###
1,25(OH)_2_ Vitamin D	0.01	###	0.03	###	−0.06	###

	α-Klotho					
	Total study patients		eGFR <60 mL/min/m^2^		eGFR ≥60 mL/min/m^2^	
	(n = 100)		(n = 70)		(n = 30)	
	r	p value	r	p value	r	p value
Age, year	0.04	###	0.07	###	0.00	###
Sex, (male = 1)	0.14	###	0.27	###	0.00	###
Body mass index	0.08	###	0.12	###	0.03	###
Systolic blood pressure	0.16	###	0.16	###	0.23	###
Diastolic blood pressure	0.18	###	0.17	###	0.27	###
Pulse rate	0.13	###	0.04	###	0.41	###
White blood cell count	0.01	###	0.02	###	−0.09	###
Hemoglobin	0.20	###	0.24	###	0.07	###
Platelet count	−0.13	###	−0.09	###	−0.28	###
Total protein	−0.06	###	−0.11	###	0.06	###
Albumin	0.19	###	0.18	###	0.16	###
Total cholesterol	0.04	###	0.05	###	−0.03	###
LDL cholesterol	0.02	###	0.08	###	−0.18	###
HDL cholesterol	0.10	###	0.05	###	0.19	###
Triglycerides	−0.05	###	0.01	###	−0.16	###
Blood urea nitrogen	−0.25	###	−0.33	###	−0.08	###
Creatinine	−0.15	###	−0.21	###	−0.16	###
eGFR	0.15	###	0.20	###	0.24	###
Alanine transaminase	0.15	###	0.22	###	−0.07	###
Aspartate transaminase	0.23	###	0.29	###	0.13	###
B-type natriuretic peptide	−0.20	###	−0.24	###	−0.07	###
C-reactive protein	−0.16	###	−0.08	###	−0.39	###
Corrected calcium	0.09	###	0.05	###	0.13	###
Inorganic phosphate	−0.17	###	−0.23	###	−0.10	###
Intact parathyroid hormone	−0.08	###	0.03	###	−0.30	###
25(OH) Vitamin D, pg/mL	0.16	###	0.10	###	0.34	###
1,25(OH)_2_ Vitamin D	0.17	###	0.17	###	0.10	###
age_2	−0.03	###	−0.03	###	−0.03	###
Sex	0.11	###	0.11	###	0.11	###
BMI	−0.20	###	−0.20	###	−0.20	###
BPs	−0.17	###	−0.17	###	−0.17	###

**Table 5:** Pearson's correlation coefficients between variable related to calcium-phosphate metabolism.

	Log(FGF23)		Log(α-Klotho)		Log(cCa)		Log(IP)		Log(iPTH)		Log(25 (OH)D)		Log(1,25 (OH)_2_D)	
	r	p value	r	p value	r	p value	r	p value	r	p value	r	p value	r	p value
Log(FGF23)	−		−0.25	0.013	−0.02	0.874	0.19	0.056	0.07	0.503	−0.03	0.759	0.01	0.917
Log(α-Klotho)	−0.25	0.013	−		0.09	0.390	−0.17	0.091	−0.08	0.428	0.16	0.114	0.17	0.083
Log(cCa)	−0.02	0.874	0.09	0.390	−		0.12	0.231	0.06	0.555	0.20	0.042	0.02	0.822
Log(IP)	0.19	0.056	−0.17	0.091	0.12	0.231	−		0.05	0.642	−0.04	0.684	−0.08	0.446
Log(iPTH)	0.07	0.503	−0.08	0.428	0.06	0.555	0.05	0.642	−		−0.09	0.359	−0.12	0.233
Log(25(OH)D)	−0.03	0.759	0.16	0.114	0.21	0.040	−0.04	0.716	−0.10	0.325	−		0.30	0.003
Log(1,25(OH)_2_D)	0.01	0.917	0.17	0.083	0.02	0.822	−0.08	0.446	−0.12	0.233	0.30	0.002	−	

### Association between calcium-phosphate metabolism-related parameters, LVEF and LVMI

**Figure 1:**
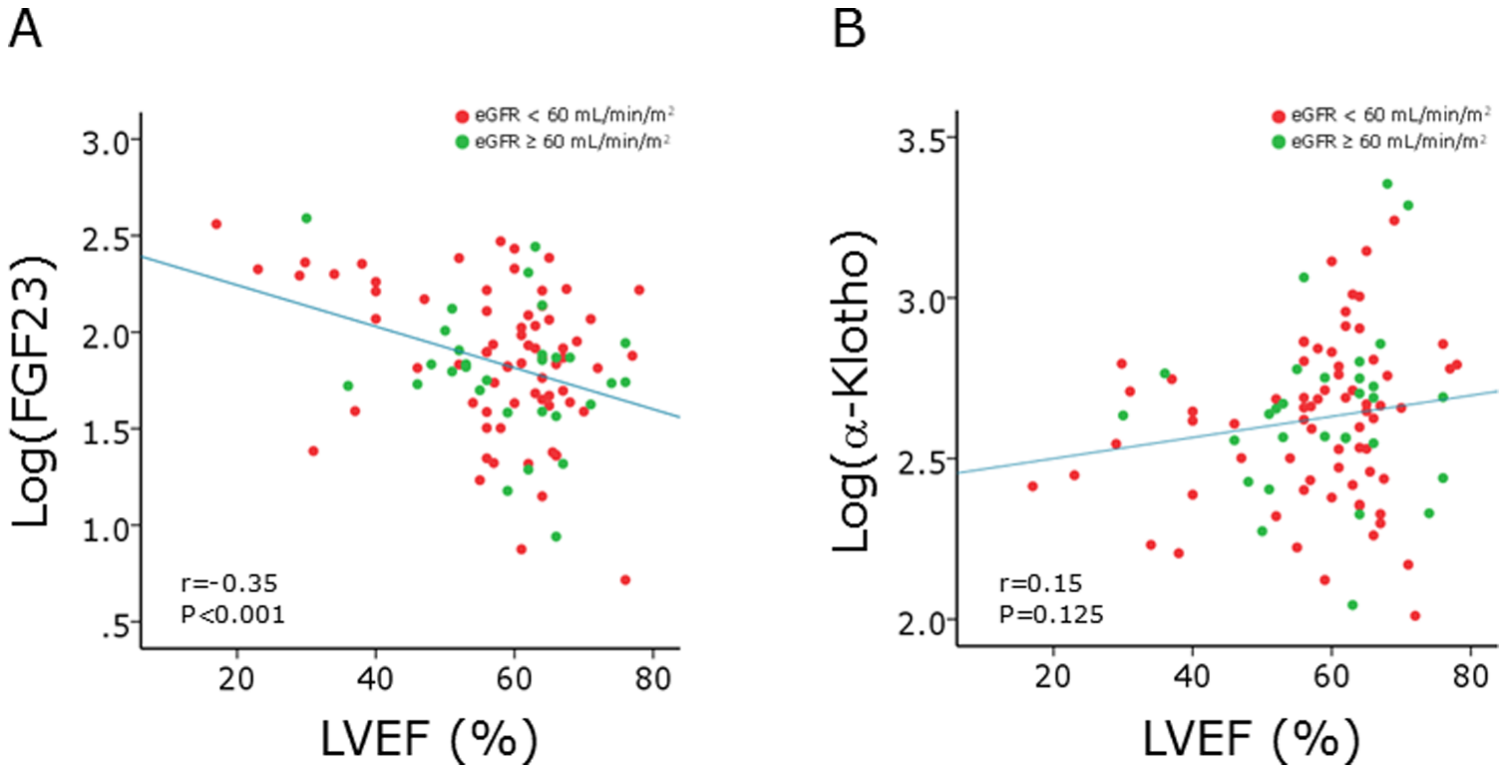
Correlation between FGF23, α-Klotho and left ventricular ejection fraction (LVEF). A. Correlation between log(FGF23) and LVEF. B. Correlation between log(α-Klotho) and LVEF. Pearson's correlation analysis was performed. Green and red closed circles indicate patients with eGFR of ≥60 mL/min/1.73 m^2^ and those with eGFR of <60 mL/min/1.73 m^2^, respectively.

**Figure 2:**
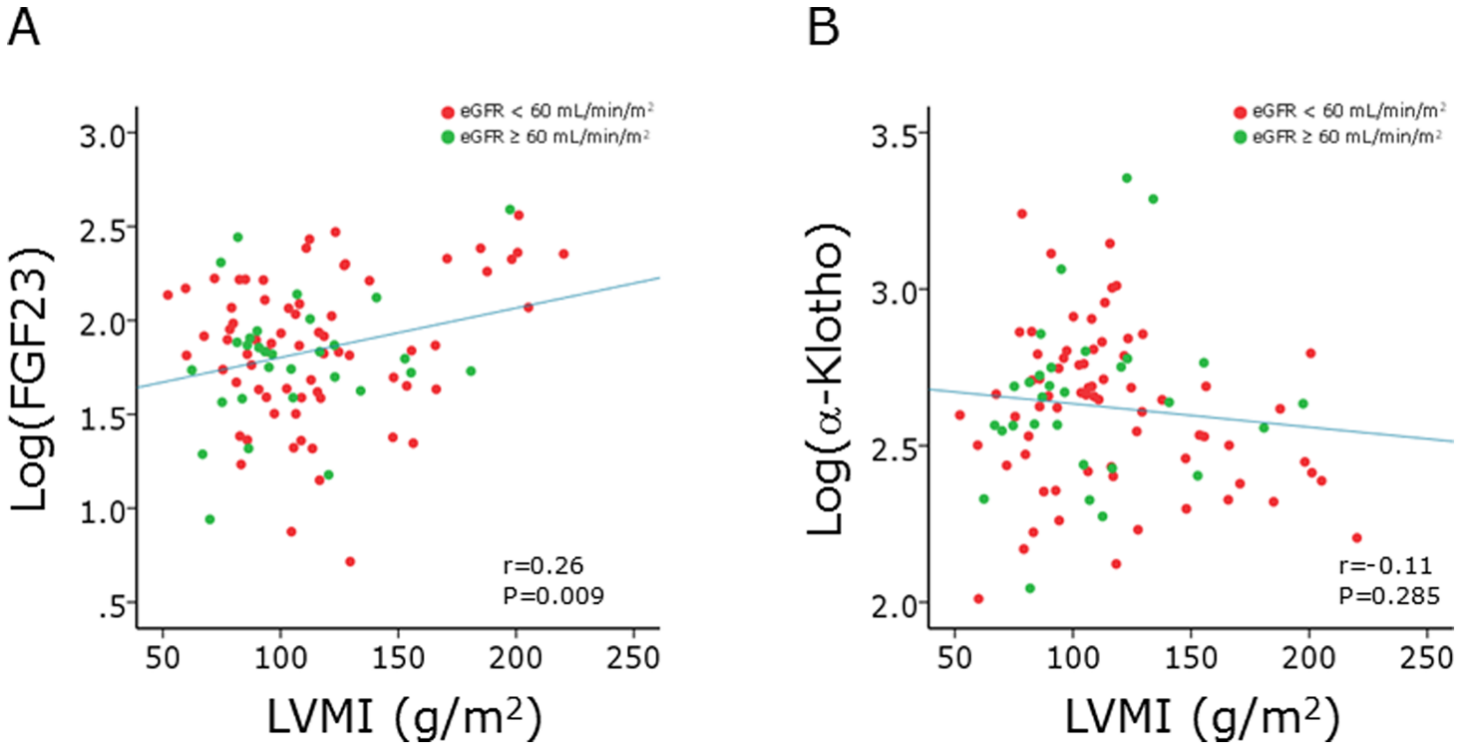
Correlation between FGF23, α-Klotho and left ventricular mass index (LVMI). A. Correlation between log(FGF23) and LVMI. B. Correlation between log(α-Klotho) and LVMI. Pearson's correlation analysis was performed. Green and red closed circles indicate patients with eGFR of ≥60 mL/min/1.73 m^2^ and those with eGFR of <60 mL/min/1.73 m^2^, respectively.

Univariate linear regression analysis showed that log(FGF23), log(iPTH), and log(25(OH)D) had significant associations with LVEF and that age, eGFR, log(FGF23), and log(iPTH) were significant associated with LVMI (Table 6). Standardized Pearson's correlation coefficients of log(FGF23) for LVEF and LVMI were −0.36 (P = 0.003) and 0.24 (P = 0.045), respectively, in 70 patients whose eGFRs were <60 mL/min/1.73m^2^, and they were −0.31 (P = 0.095) and 0.29 (P = 0.114), respectively, in 30 patients whose eGFRs were ≥60 mL/min/1.73m^2^ (Figures 1, 2). Entering all parameters used in the univariate analysis, stepwise multivariate analysis was performed. Log(FGF23) and log(25(OH)D) were selected as independent predictors for LVEF, and age, log(FGF23) and log(iPTH) were selected as independent predictors for LVMI. Relationship between FGF23, α-Klotho, low LVEF, and LV hypertrophy was also investigated by multivariate logistic regression analysis. After adjusting for age, sex, eGFR, log(iPTH), and log(25(OH)D), multivariate logistic regression analysis showed that log(FGF23) showed positive association with low LVEF (significantly) and left ventricular hypertrophy (borderline significantly) with an odds ratio of 13.46 (95% CI 1.66−109.13, p = 0.015) and 2.87 (95% CI 0.83−9.95, p = 0.097), respectively.

**Table 6:** Univariate and multivariate analysis for the association with ejection fraction and mass index of the left ventriculum.

Univariate analysis								Multivariate analysis							
	Standardized								Standardized						
Predictors	correlation coefficient		(95% CI)				P value	Selected predictors	correlation coefficient		(95% CI)				P value
Dependent variable: LVEF															
Age	−0.01	(	−0.21	-	0.19	)	0.943								
Sex (male = 1)	−0.16	(	−0.35	-	0.04	)	0.124								
Systolic BP	0.07	(	−0.13	-	0.27	)	0.490								
eGFR	0.16	(	−0.04	-	0.36	)	0.112								
log(FGF23)	−0.35	(	−0.54	-	−0.16	)	<0.001	log(FGF23)	−0.34	(	−0.52	-	−0.16	)	<0.001
log(α-Klotho)	0.15	(	−0.04	-	0.35	)	0.125								
log(cCa)	−0.06	(	−0.26	-	0.14	)	0.524								
log(IP)	0.04	(	−0.16	-	0.24	)	0.665								
log(iPTH)	−0.23	(	−0.42	-	−0.03	)	0.022								
log(25(OH)D)	0.24	(	0.04	-	0.43	)	0.018	log(25(OH)D)	0.22	(	0.04	−	0.41	)	0.018
log(1,25(OH)_2_D)	0.17	(	−0.02	-	0.37	)	0.083								
Dependent variable: LVMI															
Age	0.28	(	0.08	-	0.47	)	0.005	Age	0.29	(	0.10	-	0.47	)	0.002
Sex (male = 1)	0.02	(	−0.18	-	0.22	)	0.829								
Systolic BP	0.14	(	−0.06	-	0.34	)	0.172								
eGFR	−0.21	(	−0.41	-	−0.02	)	0.034								
log(FGF23)	0.26	(	0.07	-	0.45	)	0.009	log(FGF23)	0.25	(	0.07	-	0.43	)	0.007
log(α-Klotho)	−0.11	(	−0.31	-	0.09	)	0.285								
log(cCa)	0.12	(	−0.08	-	0.32	)	0.229								
log(IP)	−0.03	(	−0.23	-	0.17	)	0.801								
log(iPTH)	0.25	(	0.05	-	0.44	)	0.013	log(iPTH)	0.22	(	0.04	-	0.40	)	0.018
log(25(OH)D)	0.05	(	−0.16	-	0.25	)	0.655								
log(1,25(OH)_2_D)	−0.01	(	−0.21	-	0.19	)	0.903								

LVEF: left ventricular ejection fraction; LVMI; left ventricular mass index, BP; blood pressure. For multivariate analysis, stepwise regression analysis was performed by entering all the variables listed for the univariate analysis. Parameters that remained to be significant predictors are listed.

## Discussion

In the current study, we analyzed the association of circulating levels of FGF23, α-Klotho, and other calcium-phosphate metabolism-related parameters with function and mass of the left ventriculum. Univariate analysis showed that log(FGF23), but not log(α-Klotho), was associated with LVEF and LVMI. It was also found that several other calcium-phosphate metabolism-related parameters; however, after adjusting for such possible confounders, FGF23 remained to be significantly associated negatively with LVEF and positively with LV mass. To the best of our knowledge, this is the first study that targeted cardiology inpatients, namely, a high- risk population for cardiac abnormalities, for the assessment of relationships among FGF23, α-Klotho, left ventricular mass, and left ventricular function.

Several previous studies have analyzed the relationship between circulating FGF23/α-Klotho levels in the general population and in subjects with chronic kidney disease (CKD) and more advanced renal failure [26]. By measuring FGF23 levels among 659 community-dwelling women, aged 70–79 years, living in Baltimore, Dalal et al. reported that the median of FGF23 was 34.6 pg/mL [10], which was lower higher than that in our study. In addition, Westerberg et al. analyzed the FGF23 levels in a population-based cohort of Sweden that included 3014 men aged 69–81 years [27]. The median of FGF23 levels in their study was 43.5 pg/mL, which is slightly lower than that in our study. Ärnlöv et al. reported that higher serum FGF23 was associated with a significantly increased risk for cardiovascular mortality [28] and Mirza et al. reported that FGF23 was positively associated with LV mass in an elderly community population [29]. Of note, several experimental studies have suggested that FGF23 causes pathological hypertrophy of cardiomyocytes [30], which may be mediated by the FGF receptor–dependent activation of the calcineurin-NFAT signaling pathway [6]. The reason why FGF23 levels were found to be slightly higher in our study population remains unclear. On the other hand, it was reported that FGF23 levels was independently associated with LV hypertrophy [6], albeit, the results were not always uniform [31]. Thus, the relatively high FGF23 in our study subjects might be attributed to the relatively high prevalence of LV hypertrophy (36%), although ethical and racial differences should be examined in the future.

It has been reported that serum FGF23 levels were significantly elevated in CKD. In the current study, however, FGF23 levels did not significantly differ according to the two eGFR groups. Westerberg et al. reported that, by analyzing the data of adult out patients, FGF23 level was significantly elevated in those with CKD stage 4 or 5, but not in those with CKD stage 3, compared with subjects with CKD stage 1–2 [32]. We excluded patients undergoing chronic hemodialysis from the study population, and only 7 patients in the current study had eGFR of less than 30 mL/min/1.73 m^2^, which may, at least in part, explain why FGF23 level did not significantly differ between low eGFR (<60 mL/min/m^2^) and preserved eGFR (≥60 mL/min/m^2^) groups. We found that correlation between log(FGF23) and LVEF was (borderline) significant in patients with eGFRs of ≥60 mL/min/1.73 m^2^. It may suggest the possibility that FGF23 plays a role in the cardiac remodeling even in non-CKD subjects or that FGF23 is regulated by certain factors that are modulated in response to cardiac performance. Considering that FGF23 has now been considered to mediate ‘off-target,’ direct, end-organ toxicity in the heart [33], the strength of relationship between FGF23 and cardiac parameters in various CKD stages should be investigated in more detail in future studies.

The relationship between circulating α-Klotho in the general population and in patients with cardiovascular risk seems to have been less extensively assessed.

Kim et al. analyzed the α-Klotho level in patients with various renal functions among the CKD cohort [34]. They found that α-Klotho level showed graded decrease according to the CKD stage; the median of α-Klotho were 540 pg/mL in patients with CKD stage 1 and 296 pg/m in those with CKD stage 5. In addition, Kacso et al. reported that the median of α-Klotho concentrations was 400 pg/mL in diabetic patients without CKD and 800 pg/mL in controls [35]. The median of the serum α-Klotho concentrations, that was 504 pg/mL, may be said to be within a similar range of these previous studies. Semba et al. reported that higher plasma Klotho concentrations are independently associated with a lower likelihood of having cardiovascular disease in community-dwelling adults [36]. In the current study, unlike FGF23, Klotho was not associated with LVMI or LVEF, which might account for lack of reports on this topic. It should be noted, however, a recent animal experiment showed that Klotho may reduce TRPC6 channels present at the cellular surface leading to the reduction of the inward calcium current under certain condition of stress, resulting in protecting the heart from hypertrophy and systolic dysfunction [37].

In the current study, multivariate analysis showed that 25(OH)D and iPTH were positively associated with LVEF and LVMI, respectively, findings that were in accordance with previous observations [38], [39], although such associations may depend on the target population [40]. Considering that LV hypertrophy and reduced LVEF are parameters predictive of poor cardiovascular outcome [41], and that both circulating levels of FGF23 and α-Klotho are regulated in patients with chronic kidney disease or advanced renal failure [42], alterations in the circulating levels or activity of FGF23/α-Klotho may link the chronic kidney disease with higher cardiovascular risk [43]. Circulating levels of Ca, IP, and 25(OH)D were also reported to be associated with cardiovascular risk [44], [45] and elevated PTH levels were associated with left ventricular mass and severity of heart failure [39], [46]; therefore, one should confirm, as has been done in the current study, whether the observed relationship between FGF23/α-Klotho and cardiac abnormality is mediated or confounded by the above-mentioned variables, whose levels are related to FGF23/α-Klotho levels [47], [48].

There are several limitations in the current study. First, the current study included patients with various cardiovascular diseases. The relationship between FGF23 and cardiac remodeling might differ according the etiology of the background disorders. Second, although exposure of primary cardiomyocytes to FGF23 was reported to result in elevated intracellular calcium and increased ventricular muscle strip contractility in the experimental model [30], whether FGF23 truly represents a useful biomarker, or is a simple bystander, for cardiac dysfunction and hypertrophy should be analyzed in the longitudinal studies. In addition, whether life-style modifications or drug interventions that might reduce circulating FGF23 levels would improve the cardiovascular prognosis should be investigated.

In summary, by analyzing data from cardiology inpatients, we found that circulating levels of FGF23 were positively associated with LV mass, LV hypertrophy, reduced LV systolic function, and plasma BNP concentration. This relationship was statistically significant after adjustment for other calcium-phosphate metabolism-related parameters including α-Klotho. Whether modulation of FGF23 activity would improve cardiac outcome in such a high-risk population awaits further investigation.
