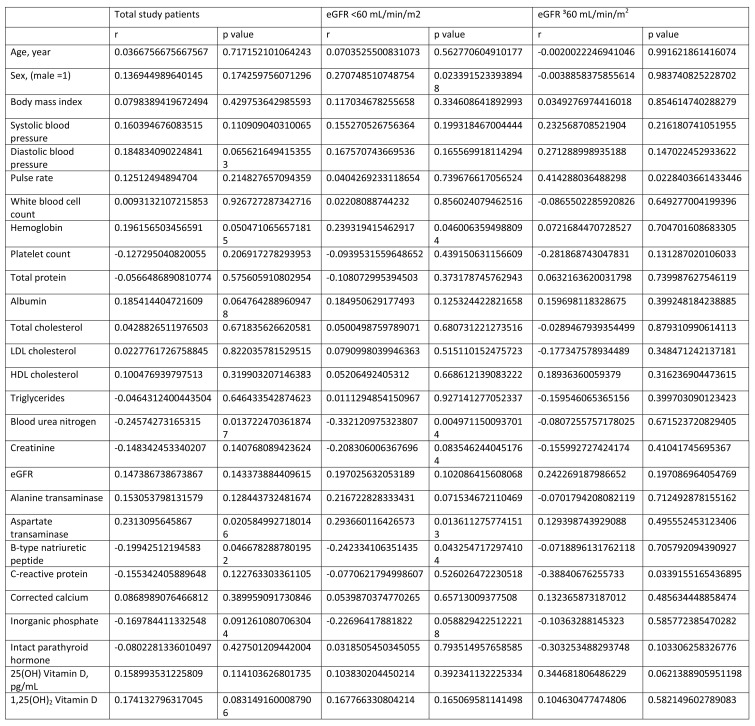# Correction: Association between Circulating Fibroblast Growth Factor 23, α-Klotho, and the Left Ventricular Ejection Fraction and Left Ventricular Mass in Cardiology Inpatients

**DOI:** 10.1371/annotation/13086f84-18f2-4ea3-881a-0484b0c411ee

**Published:** 2013-10-25

**Authors:** Kensaku Shibata, Shu-ichi Fujita, Hideaki Morita, Yusuke Okamoto, Koichi Sohmiya, Masaaki Hoshiga, Nobukazu Ishizaka

Table 3 and Table 4 were compiled into one image. The values under the "P-value" columns in Table 3 and Table 4 are also missing.

Please see the corrected Table 3, with the heading "FGF23", here: 

**Figure:**
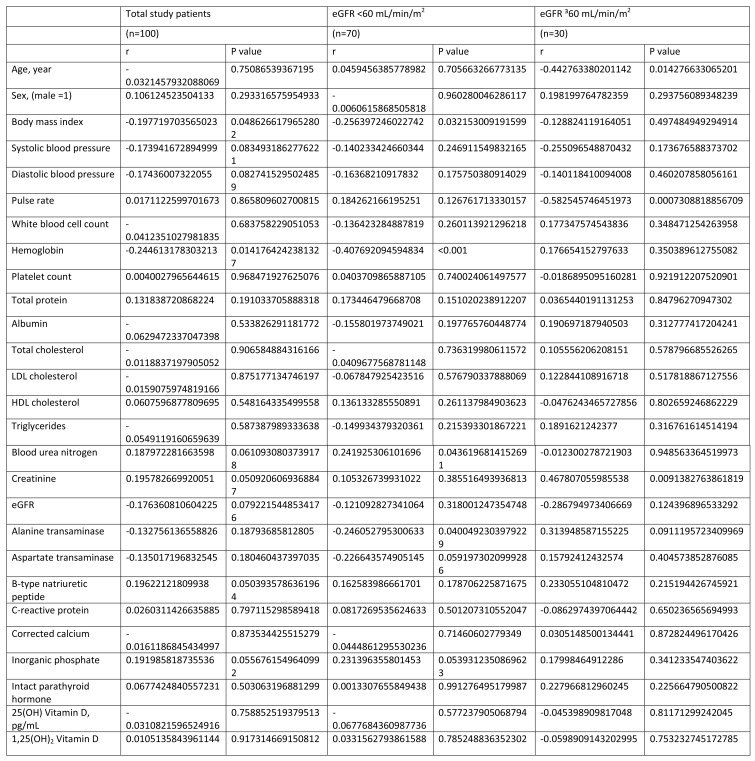


Please see the corrected Table 4, with the heading "alpha-Klotho", here: 

**Figure:**