# Correction: Association between Circulating Fibroblast Growth Factor 23, α-Klotho, and the Left Ventricular Ejection Fraction and Left Ventricular Mass in Cardiology Inpatients

**DOI:** 10.1371/journal.pone.0093346

**Published:** 2014-04-01

**Authors:** The PLOS ONE Staff

In Table 1, values for Sinus rhythm and Pace maker rhythm in the Cardiac rhythm section are incorrect. The correct version of Table 1 can be viewed here.

**Table 1:** Demographic characteristics of the study patients.

	Total study patients	eGFR <60 mL/min/m^2^	eGFR ≥60 mL/min/m^2^	
Variables	(n = 100)	(n = 70)	(n = 30)	P value
Clinical characteristics				
Age	65.4±13.0	68.4 ±10.9	58.3 ±14.9	<0.001
Sex (women/men)	27/73	10 /60	17 /13	<0.001
Body mass index, kg/m^2^	23.1±3.1	23.2 ±3.0	23.0 ±3.2	0.740
Systolic blood pressure, mmHg	126.1 ±19.7	126.5 ±19.6	125.1 ±20.1	0.744
Diastolic blood pressure, mmHg	71.6 ±12.4	70.9 ±12.0	73.3 ±13.2	0.364
Pulse rate, bpm	72.3 ±13.7	72.6 ±14.9	71.4 ±10.5	0.695
Past History				
previous PCI	44(44.0 )	33(47.1 )	11(36.7 )	0.333
previous CABG	7(7.0 )	4(5.7 )	3(10.0 )	0.441
Cardiovascular disease				
Ischemic heart disease, n (%)	59 (59.0 )	43 (61.4 )	16 (53.3 )	0.451
Cardiomyopathy, n (%)	10 (10.0 )	7 (10.0 )	3 (10.0 )	1.000
NYHA class III/IV, n (%)	12 (12.0 )	5 (7.1 )	7 (23.3 )	0.022
Aortic aneurysm, n (%)	6 (6.0 )	5 (7.1 )	1 (3.3 )	0.462
Arrythmia, n (%)	22 (22.0 )	12 (17.1 )	10 (33.3 )	0.073
Peripheral artery disease, n (%)	8 (8.0 )	7 (10.0 )	1 ( 3.3 )	0.260
Valvular heart disease, n (%)	8 ( 8.0 )	7 ( 10.0 )	1 ( 3.3 )	0.260
Cardiac rhythm				
Sinus rhythm, n (%)	78 ( 78.0 )	56 ( 80.0 )	22 ( 73.3 )	0.687
Atrial fibrillation, n (%)	15 ( 15.0 )	10 ( 14.3 )	5 ( 16.7 )	
Pace maker rhythm, n (%)	7 ( 7.0 )	4 ( 5.7 )	3 ( 10.0 )	
Smoking status				
Never, n (%)	33 ( 33.0 )	24 ( 34.3 )	9 ( 30.0 )	0.658
Former, n (%)	50 ( 50.0 )	33 ( 47.1 )	17 ( 56.7 )	
Current, n (%)	17 ( 17.0 )	13 ( 18.6 )	4 ( 13.3 )	
Medication				
ACE inhibitors/ARB, n (%)	51 ( 51.0 )	41 ( 58.6 )	10 ( 33.3 )	0.021
Beta blockers, n (%)	38 ( 38.0 )	32 ( 45.7 )	6 ( 20.0 )	0.015
Calcium channel blockers, n (%)	44 ( 44.0 )	35 ( 50.0 )	9 ( 30.0 )	0.065
Aldosterone antagonist, n (%)	13 ( 13.0 )	11 ( 15.7 )	2 ( 6.7 )	0.218
Diuretics				
Loop, n (%)	18 ( 18.0 )	15 ( 21.4 )	3 ( 10.0 )	0.173
Thiazide, n (%)	5 ( 5.0 )	4 ( 5.7 )	1 ( 3.3 )	0.617
Antidiabetic drugs				
Sulfonylurea, n (%)	10 ( 10.0 )	7 ( 10.0 )	3 ( 10.0 )	1.000
DPP4 inhibitors, n (%)	9 ( 9.0 )	6 ( 8.6 )	3 ( 10.0 )	0.819
Insulin, n (%)	4 ( 4.0 )	2 ( 2.9 )	2 ( 6.7 )	0.373
Others, n (%)	8 ( 8.0 )	4 ( 5.7 )	4 ( 13.3 )	0.198
Lipid lowering drugs				
Statin, n (%)	52 ( 52.0 )	39 ( 55.7 )	13 ( 43.3 )	0.256
Fibrate, n (%)	1 ( 1.0 )	0 ( 0.0 )	1 ( 3.3 )	0.125
Others, n (%)	7 ( 7.0 )	6 ( 8.6 )	1 ( 3.3 )	0.347

PCI, percutaneous coronary intervention; coronary artery bypass graft; ACE, angiotensin converting enzyme; ARB, angiotensin II receptor blocker; DPP4, di-peptidyl peptidase-4. P values are meant to the comparison between eGFR ≥60 mL/min/m^2^ and <60 mL/min/m^2^ groups.

The corrected versions of Tables 3 and 4 are also incorrect. Additional significant figures were added during the correction of these tables. The publisher apologizes for these errors. The correct versions of Tables 3 and 4 can be viewed here.

**Table 3:** Spearman's correlation coefficients between FGF23 and various variables.

	FGF23					
	Total study patients		eGFR <60 mL/min/m^2^		eGFR ≥60 mL/min/m^2^	
	(n = 100)		(n = 70)		(n = 30)	
	r	P value	r	P value	r	P value
Age, year	-0.03	0.751	0.05	0.706	-0.44	0.014
Sex, (male = 1)	0.11	0.293	-0.01	0.960	0.20	0.294
Body mass index	-0.20	0.049	-0.26	0.032	-0.13	0.497
Systolic blood pressure	-0.17	0.083	-0.14	0.247	-0.26	0.174
Diastolic blood pressure	-0.17	0.083	-0.16	0.176	-0.14	0.460
Pulse rate	0.02	0.866	0.18	0.127	-0.58	0.001
White blood cell count	-0.04	0.684	-0.14	0.260	0.18	0.348
Hemoglobin	-0.24	0.014	-0.41	<0.001	0.18	0.350
Platelet count	0.00	0.968	0.04	0.740	-0.02	0.922
Total protein	0.13	0.191	0.17	0.151	0.04	0.848
Albumin	-0.06	0.534	-0.16	0.198	0.19	0.313
Total cholesterol	-0.01	0.907	-0.04	0.736	0.11	0.579
LDL cholesterol	-0.02	0.875	-0.07	0.577	0.12	0.518
HDL cholesterol	0.06	0.548	0.14	0.261	-0.05	0.803
Triglycerides	-0.05	0.587	-0.15	0.215	0.19	0.317
Blood urea nitrogen	0.19	0.061	0.24	0.044	-0.01	0.949
Creatinine	0.20	0.051	0.11	0.386	0.47	0.009
eGFR	-0.18	0.079	-0.12	0.318	-0.29	0.124
Alanine transaminase	-0.13	0.188	-0.25	0.040	0.31	0.091
Aspartate transaminase	-0.14	0.180	-0.23	0.059	0.16	0.405
B-type natriuretic peptide	0.20	0.050	0.16	0.179	0.23	0.215
C-reactive protein	0.03	0.797	0.08	0.501	-0.09	0.650
Corrected calcium	-0.02	0.874	-0.04	0.715	0.03	0.873
Inorganic phosphate	0.19	0.056	0.23	0.054	0.18	0.341
Intact parathyroid hormone	0.07	0.503	0.00	0.991	0.23	0.226
25(OH) Vitamin D, pg/mL	-0.03	0.759	-0.07	0.577	-0.05	0.812
1,25(OH)_2_ Vitamin D	0.01	0.917	0.03	0.785	-0.06	0.753

**Table 4:** Spearman’s correlation coefficients between α-Klotho and various variables.

	α-Klotho					
	Total study patients		eGFR <60 mL/min/m^2^		eGFR ≥60 mL/min/m^2^	
	(n = 100)		(n = 70)		(n = 30)	
	r	P value	r	P value	r	P value
Age, year	0.04	0.717	0.07	0.563	0.00	0.992
Sex, (male = 1)	0.14	0.174	0.27	0.023	0.00	0.984
Body mass index	0.08	0.430	0.12	0.335	0.03	0.855
Systolic blood pressure	0.16	0.111	0.16	0.199	0.23	0.216
Diastolic blood pressure	0.18	0.066	0.17	0.166	0.27	0.147
Pulse rate	0.13	0.215	0.04	0.740	0.41	0.023
White blood cell count	0.01	0.927	0.02	0.856	-0.09	0.649
Hemoglobin	0.20	0.050	0.24	0.046	0.07	0.705
Platelet count	-0.13	0.207	-0.09	0.439	-0.28	0.131
Total protein	-0.06	0.576	-0.11	0.373	0.06	0.740
Albumin	0.19	0.065	0.18	0.125	0.16	0.399
Total cholesterol	0.04	0.672	0.05	0.681	-0.03	0.879
LDL cholesterol	0.02	0.822	0.08	0.515	-0.18	0.348
HDL cholesterol	0.10	0.320	0.05	0.669	0.19	0.316
Triglycerides	-0.05	0.646	0.01	0.927	-0.16	0.400
Blood urea nitrogen	-0.25	0.014	-0.33	0.005	-0.08	0.672
Creatinine	-0.15	0.141	-0.21	0.084	-0.16	0.410
eGFR	0.15	0.143	0.20	0.102	0.24	0.197
Alanine transaminase	0.15	0.128	0.22	0.072	-0.07	0.712
Aspartate transaminase	0.23	0.021	0.29	0.014	0.13	0.496
B-type natriuretic peptide	-0.20	0.047	-0.24	0.043	-0.07	0.706
C-reactive protein	-0.16	0.123	-0.08	0.526	-0.39	0.034
Corrected calcium	0.09	0.390	0.05	0.657	0.13	0.486
Inorganic phosphate	-0.17	0.091	-0.23	0.059	-0.10	0.586
Intact parathyroid hormone	-0.08	0.428	0.03	0.794	-0.30	0.103
25(OH) Vitamin D, pg/mL	0.16	0.114	0.10	0.392	0.34	0.062
1,25(OH)_2_ Vitamin D	0.17	0.083	0.17	0.165	0.10	0.582
